# Adverse Outcome of a Solitary Fibrous Tumor Originating in the Bladder

**DOI:** 10.1002/iju5.70013

**Published:** 2025-03-11

**Authors:** Takato Nishino, Masaki Shimbo, Eri Fukagawa, Kazutaka Narimoto, Jun Hashimoto, Shin Ogita, Naoki Kanomata, Kazunori Hattori, Fumiyasu Endo

**Affiliations:** ^1^ Department of Urology St. Luke's International Hospital Chuo‐ku Japan; ^2^ Department of Medical Oncology St. Luke's International Hospital Chuo‐ku Japan; ^3^ Department of Pathology St. Luke's International Hospital Chuo‐ku Japan

**Keywords:** metastases, poor prognosis, robot‐assisted surgery, solitary fibrous tumor, urinary bladder

## Abstract

**Introduction:**

Solitary fibrous tumors originating in the bladder are extremely rare. While generally associated with favorable outcomes, some show invasive behavior. We report a case of a solitary fibrous tumor originating in the bladder that was treated with multimodal therapy.

**Case Presentation:**

A 68‐year‐old male presented with urinary retention. Imaging revealed a well‐defined 6.0 cm mass compressing the prostate. A biopsy suggested stromal sarcoma. Robot‐assisted cystoprostatectomy was performed. Pathological examination revealed a solitary fibrous tumor originating from the bladder invading the prostate. Despite negative margins, lung nodules and a pelvic mass appeared 43 months postoperatively. Initially, these were treated with pazopanib, followed by doxorubicin and eribulin due to disease progression. The patient eventually transitioned to palliative care and passed away 69 months after diagnosis.

**Conclusion:**

There are no effective systemic treatments for solitary fibrous tumors, which can lead to poor outcomes. Individualized treatment approaches are necessary.

**Summary**

- Solitary fibrous tumors (SFTs) of the bladder are rare. Surgical resection is the primary treatment and generally has favorable outcomes, but we report a rare case of SFT with recurrence and metastasis 3 years after surgery, leading to a poor outcome. This case highlights the importance of long‐term follow‐up and aggressive management for recurrent SFTs.

**Abbreviations**
CT: computed tomography
PSA: prostate‐specific antigen
PSS: prostate stromal sarcoma
SFT: solitary fibrous tumor

## 1 Introduction

Solitary fibrous tumors (SFTs) are rare mesenchymal neoplasms. The prognosis is generally favorable, and bladder origin is rare [1, 2]. We report an SFT originating in the bladder that required multimodal treatment and had a fatal outcome.

## 2 Case Presentation

**FIGURE 1:**
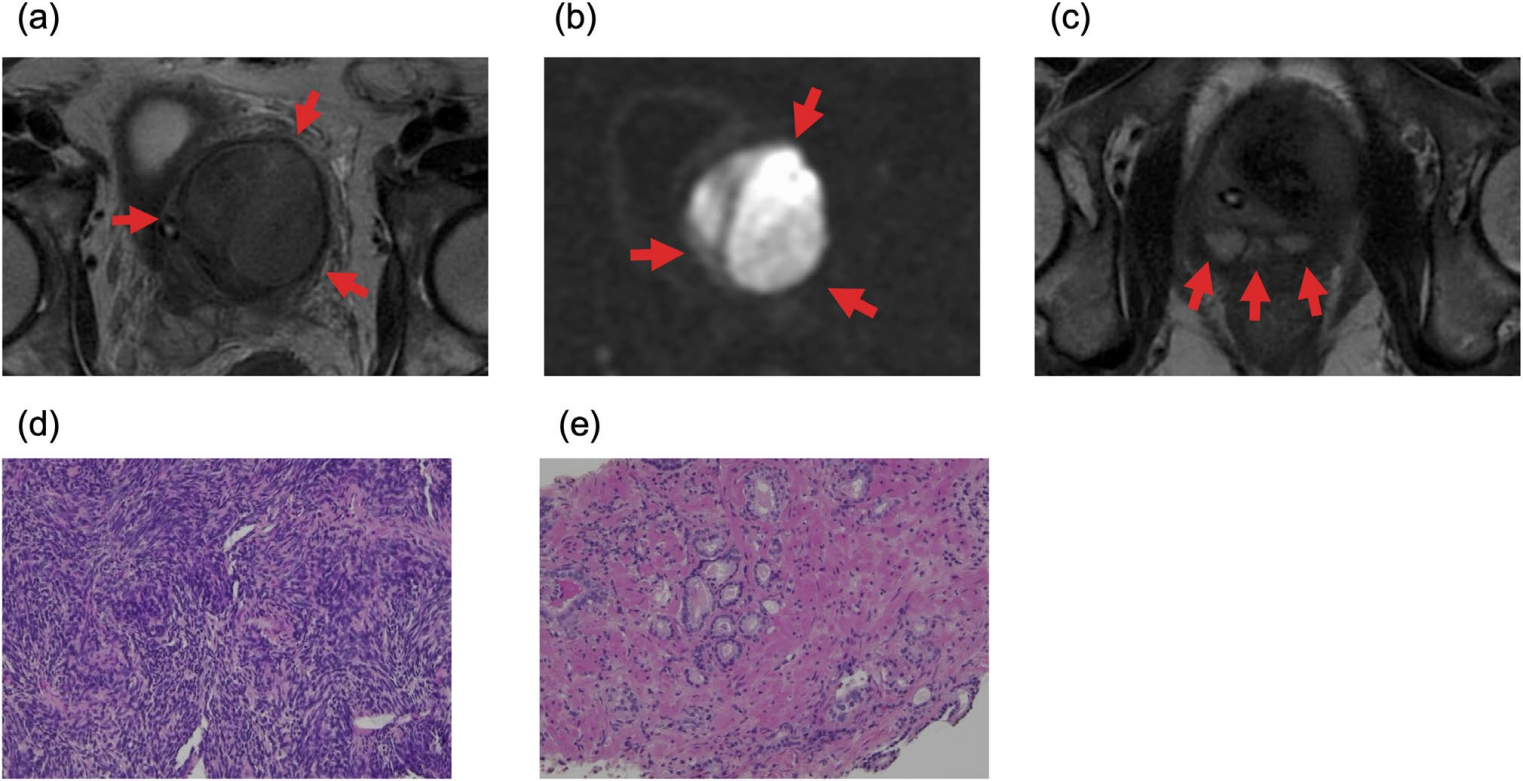
(a) Magnetic resonance imaging revealed a 6.0‐cm tumor. On T2‐weighted images, the tumor showed heterogeneous high signal intensity. (b) Diffusion‐weighted images had a pronounced high intensity. (c) The tumor displaced the normal prostate to the right caudal side. (d) A transperineal biopsy and histological examination were performed. The biopsy specimen showed spindle cells with pleomorphic atypical nuclei proliferating in a fascicular pattern and high cellularity (hematoxylin and eosin). (e) Biopsy specimen of the prostate revealed incidental prostate cancer, International Society of Urological Pathology Grade Group 2 (hematoxylin and eosin).

A 68‐year‐old man presented with urinary retention, weight loss, and perineal pain. His medical history included a stroke at age 63 without residual deficits, hypertension, and diabetes mellitus. Routine blood tests were normal, and prostate‐specific antigen (PSA) was 2.47 ng/mL. Pelvic magnetic resonance imaging revealed a 6.0‐cm irregular mass with well‐defined boundaries. The tumor showed heterogeneous high signal intensity on T2‐weighted images and marked high signal on diffusion‐weighted images (Figure 1a–c). The tumor displaced the normal prostate to the right caudal side. Enhanced computed tomography (CT) revealed no signs of lymph node or distant metastasis. A transperineal biopsy revealed spindle cells with pleomorphic atypical nuclei proliferating in a fascicular pattern and high cellularity (Figure 1d). No epithelial components were observed. The findings suggested a prostate stromal sarcoma (PSS). Incidental prostate cancer, International Society of Urological Pathology Grade Group 2, was diagnosed (Figure 1e).

**FIGURE 2:**
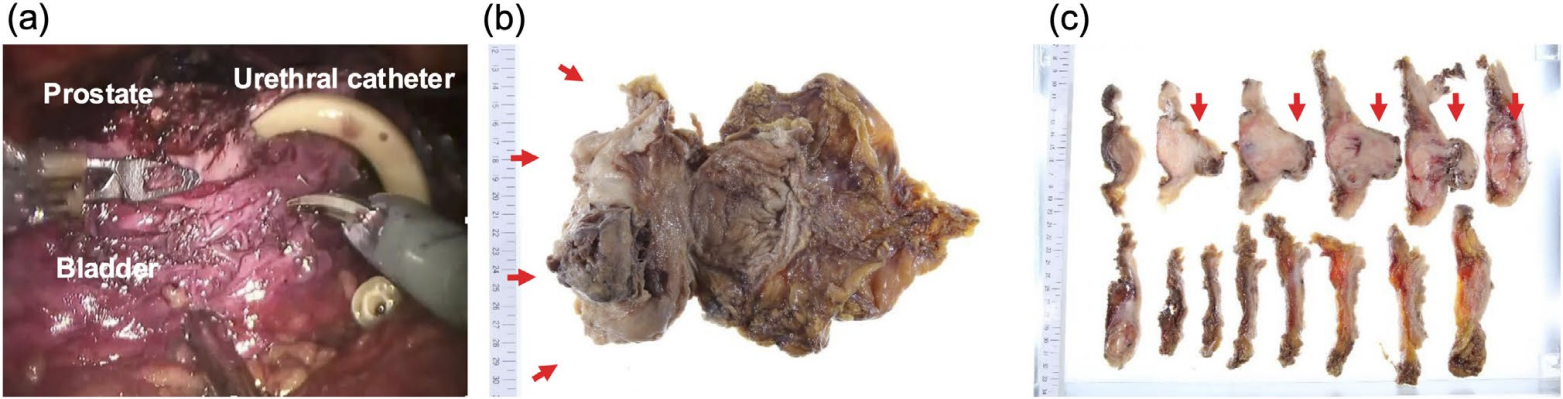
(a) Robot‐assisted radical cystoprostatectomy was performed. The tumor was strongly adherent to the surrounding tissues. Retrograde dissection was performed after urethral transection. The tumor was resected en bloc with the bladder and prostate. (b, c) Grossly, the tumor was a grayish, firm, solid mass measuring 6.0 × 5.0 × 4.5 cm. The tumor originated from the left bladder wall, infiltrating the bladder cavity and prostate (pT4a).

PSS is an aggressive tumor, and complete surgical resection is the primary treatment. As tumor resection appeared achievable with total cystoprostatectomy, we prioritized surgical treatment. Considering the aggressive nature of the tumor, pelvic lymph node dissection was also performed. Regarding the surgical technique, robot‐assisted tumor resection has already been reported for PSS [3], and we selected a robot‐assisted approach. Urinary diversion was performed by constructing an ileal conduit. The tumor was strongly adherent to the surrounding tissues, resulting in marked bleeding during the dissection. A posterolateral approach failed to progress, prompting retrograde dissection following urethral transection (Figure 2a). The postoperative course was uneventful.

**FIGURE 3:**
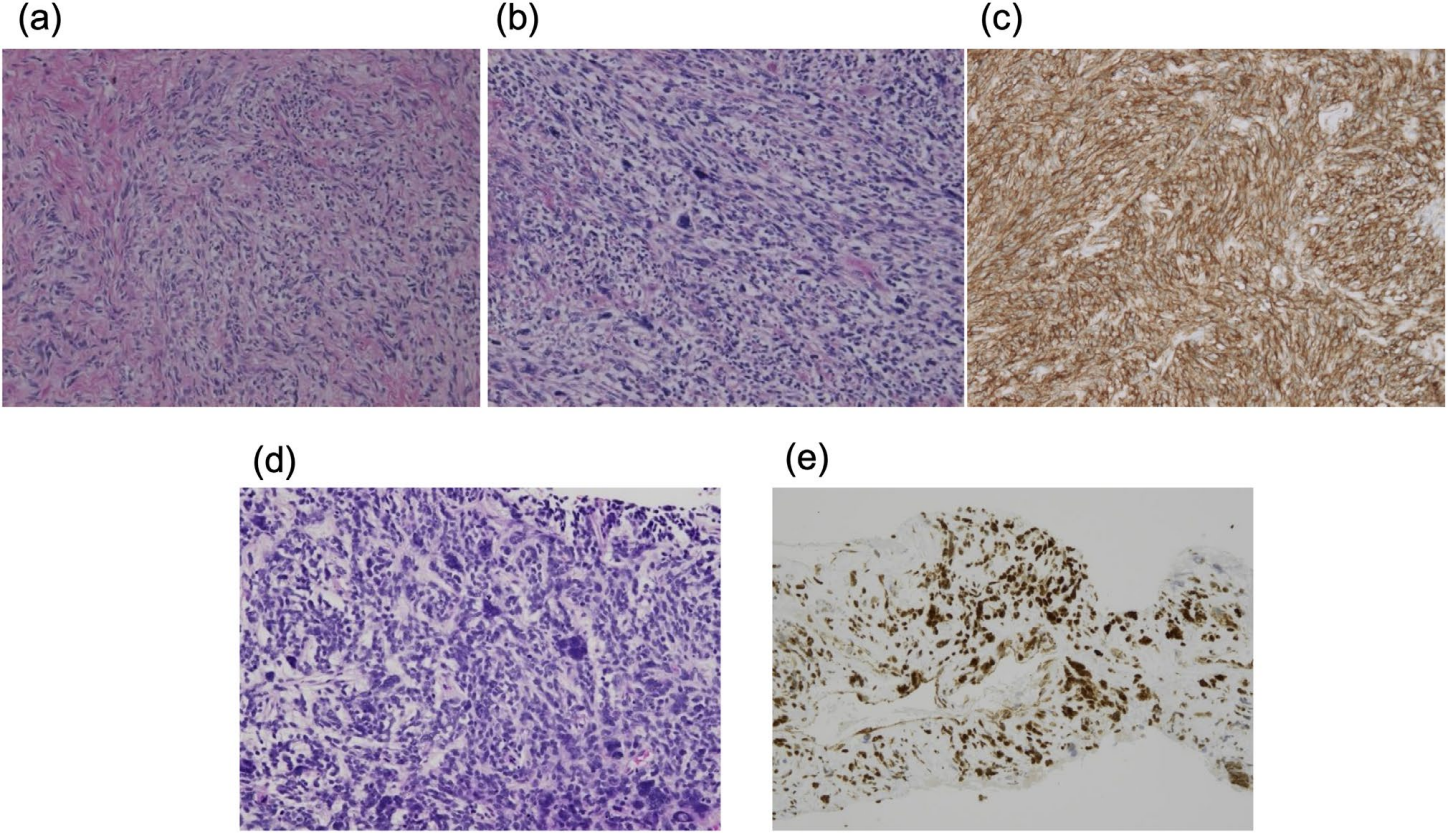
There were spindle cells within fine fibrous stroma, and hyalinized staghorn‐shaped blood vessels. The tumor was positive for CD34 and STAT6. (a)–(c) Specimen of the bladder tumor. (a) Hematoxylin and eosin. (b) Hematoxylin and eosin. (c) CD34. (d, e) Specimen of lung metastasis: (d) Hematoxylin and eosin, and (e) STAT6.

Pathological examination confirmed the diagnosis of bladder SFT. The tumor was a grayish, firm, solid mass originating from the left bladder wall, infiltrating the bladder cavity and prostate (Figure 2b,c). Histologically, the tumor consisted of spindle cells with pale to lightly eosinophilic cytoplasm within a fine fibrous stroma (Figure 3a–c). Branching, hyalinized staghorn‐shaped blood vessels were prominent. Some regions exhibited increased cellular density and marked nuclear pleomorphism. Approximately 20 mitoses per 10 high‐power fields and necrotic changes were observed. The tumor was staged as pT4bN0 according to the Union for International Cancer Control, 8th Edition [4]. Complete surgical resection with negative margins was achieved. Immunohistochemically, the tumor was positive for CD34, vimentin, and STAT6, and negative for α‐SMA, S100, c‐kit, and desmin (Figure 3d,e).

The surgery achieved R0 resection, and the multidisciplinary team chose observation without adjuvant therapy due to the generally benign nature of SFTs. However, increased mitotic activity and tumor necrosis raised concerns, prompting the need for long‐term follow‐up. At 43 months, CT revealed nodules in the right lung and a pelvic mass, with undetectable PSA levels. These findings were considered metastases and recurrence of SFT. Given the patient's general condition, surgical resection was deemed unfeasible, leading to the initiation of systemic therapy.

**FIGURE 4:**
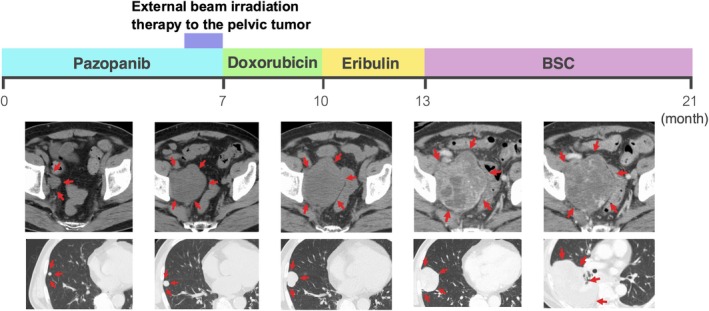
The treatment course following recurrence. Systemic pazopanib, doxorubicin, and eribulin were administered, none of which achieved notable efficacy. The tumor continued to progress, and the patient died 69 months after diagnosis.

Figure 4 shows the course of treatment following recurrence. The first‐line treatment with pazopanib was ineffective, leading to increased pelvic tumor size and associated pain and right leg numbness. Palliative external beam irradiation therapy (50Gy/25fr) was administered to the pelvic tumor. This was followed by the second‐line treatment with doxorubicin, which had limited efficacy. The third‐line treatment with eribulin was also ineffective. The lung metastasis was re‐biopsied, and molecular profiling was performed. TERT and TP53 loss mutations were identified, with no actionable targets. The patient's condition deteriorated, necessitating a transition to palliative care. He died 69 months after the initial diagnosis.

## 3 Discussion

The standard treatment for SFTs is complete surgical resection. While the majority of SFTs progress slowly, some cases may experience local recurrence or metastasis, even up to 20 years after resection [5].

Evidence for medical therapy in SFT is limited. In this case, pazopanib [6] and doxorubicin [7] were selected based on reports suggesting their efficacy in SFT. Due to his cardiovascular risk, caution was warranted in using the cardiotoxic agent doxorubicin; therefore, pazopanib—generally better tolerated—was chosen as the first‐line treatment. Unfortunately, no response was observed, and for the third‐line treatment, eribulin was chosen. SFT is a tumor characterized by the NAB2‐STAT6 fusion gene [8], and its pathogenesis differs from that of conventional soft tissue sarcomas. Ideally, treatment strategies should be based on evidence specific to SFT; however, because therapeutic options remain limited, we selected a regimen based on retrospective studies in soft tissue sarcoma treatment [9]. Regarding radiation therapy, SFT is generally considered to be a radioresistant tumor, and its efficacy is limited. A retrospective study reported an objective response rate of 38% for palliative radiation therapy in metastatic SFT [10]. In this case, radiation therapy was added in anticipation of improving right lower limb symptoms. Considering the tumor's radioresistance, a total dose of 50 Gy was chosen. The pelvic location of the tumor and potential impacts on adjacent organs were also considered, leading to the decision to use fractionated radiation therapy.

The prognosis of SFTs is variable. A variety of risk assessment models, such as the Demicco model, which evaluates age at presentation, tumor size, mitotic count, and necrosis, are used to predict outcomes [11, 12, 13]. In the original Demicco model [11], this case is classified as intermediate risk with a total score of 4 points: 1 point for age, 1 point for tumor size, and 2 points for mitotic count. Intermediate‐risk cases are associated with a 5‐year metastasis‐free survival rate of 77% and a 5‐year disease‐specific survival rate of 93%. However, bladder SFTs are rare even among SFT cases, and caution is necessary when applying these models to predict outcomes for such cases.

Of the approximately 30 reported cases of bladder SFT, although most patients experience favorable outcomes following surgical resection, there have been reports of three cases of local recurrence [14] and one case of metastatic recurrence resulting in death [15]. Bladder SFTs often remain asymptomatic, potentially delaying detection; indeed, 11 cases were discovered when the tumor measured 10 cm or larger, while only 5 were identified when smaller than 5 cm [15]. Consequently, bladder SFTs may be associated with relatively worse oncologic outcomes. Conversely, some reports indicate that SFTs arising in the extremities—typically detected earlier and more amenable to complete resection—exhibit a higher rate of metastatic recurrence compared to tumors at other sites [16], underscoring the current challenge in predicting prognosis based solely on the primary location.

This case highlights the aggressive potential of bladder SFT. Despite achieving an R0 resection, the patient experienced recurrence and metastasis within a few years. This case highlights the need for long‐term monitoring and aggressive treatment.

## Disclosure

Approval of the research protocol by an Institutional Review Board and the approval number: N/A.

Registry and the Registration No. of the study/trial: N/A.

## Consent

Informed consent was obtained from the subject.

## Conflicts of Interest

The authors declare no conflicts of interest.
